# Ipsis Consuescere Rebus

**Published:** 1886-05

**Authors:** 


### IPSIS CONSUESCERE REBUS.

We earnestly hope that the suggestion with reference to the appointment of a Committee on Pathological Specimens, made to the CHICAGO MEDICAL SOCIETY by Professor Charles T. Parkes, on retiring from the presidency a few evenings since, will soon receive serious attention.

The importance "of acquainting ourselves, by means of our senses, with the forms, properties and changes of things, in order that the language we employ may, as far as possible, be employed INTUITIVELY," cannot be exaggerated. It is needful to bear in mind Bacon's advice—*ipsis consuescere rebus—to accustom ourselves to things themselves.*

The late PROFESSOR JEVONS was in the habit of emphasizing the following paragraph, taken from his "Lessons in Logic:"

"There is no worse habit for a student or reader to acquire than that of accepting words instead of a knowledge of things. It is perhaps worse than useless to read a work on natural history about Infusoria, Foraminifera, Rotifera and the like, if these names do not convey clear images to the mind. Nor can a student who has not witnessed experiments, and examined the substances with his own eyes, derive any considerable advantage from works on chemistry and natural philosophy, where he will meet with hundreds of new terms which would be to him mere empty and confusing signs."

The supply of material for pathologico-anatomical research in Chicago is ample. DR. ELBERT WING, Pathologist to the Cook County Hospital, has expressed his desire to place at the disposal of the SOCIETY the interesting specimens which are inspected at the dead-house.

